# Psychometric properties of the Persian version of the workplace fun scale among nurses: a cross-sectional study

**DOI:** 10.1038/s41598-024-77304-x

**Published:** 2024-10-29

**Authors:** Azam Hashemian Moghadam, Reza Imashi, Roghayeh Yaghoobi Saghezchi, Alireza Mirzaei

**Affiliations:** 1 Department of Psychology, Faculty of Education and Psychology, Ferdowsi University of Mashhad, Mashhad, Iran; 2 Department of Emergency Nursing, School of Nursing and Midwifery, Ardabil University of Medical Sciences, Ardabil, Iran; 3 Department of Emergency Medicine, School of Medicine, Ardabil University of Medical Sciences, Ardabil, Iran

**Keywords:** Workplace fun, Scale, Validity and reliability, Confirmatory factor analysis, Nurse, Health occupations, Health services, Medical ethics, Occupational health, Public health, Quality of life

## Abstract

Previous research has highlighted the importance of workplace fun in enhancing employee satisfaction and performance, particularly in high-stress professions like nursing. However, a notable gap exists in understanding how workplace fun is perceived and measured among nurses in Persian-speaking countries. This study addresses this gap by translating the Workplace Fun Scale and assessing its psychometric properties among nurses. The findings will provide insights into the scale’s applicability in these contexts and pave the way for healthcare organizations to significantly explore ways to enhance nurses’ enjoyment of work in Persian-speaking countries. This survey, which involved 321 nurses from medical education centers in Ardabil, was conducted with a meticulous and rigorous methodology. Standard questionnaires collected the data, including a demographic form and the translated Workplace Fun Scale. The study examined the three aspects of workplace fun: fun activities, coworker socializing, and manager support for fun. Confirmatory factor analysis was used to validate the structure, and reliability was assessed through retest coefficients, Cronbach’s alpha coefficients, and composite reliability coefficients. The data were analyzed using SPSS version 14 and LISREL version 8.8, ensuring the highest research standards. The validity of both form and content was confirmed through translation and reverse translation. The Workplace Fun Scale showed high internal consistency and reliability, with significant Cronbach’s alpha coefficients, composite reliability, and two-week retest coefficients of 0.859, 0.885, and 0.459, respectively (all at the *p* < 0.01 level). Fit indices, including GFI (0.97), AGFI (0.94), CFI (0.99), NFI (0.98), TLI (0.97), and SRMR (0.04), indicated a good fit of the measurement model, confirming the validity of the scale in assessing workplace fun. The confirmatory factor analysis results indicated that the translated version of the workplace fun scale, adapted from Tews, exhibited a robust factor structure and internal homogeneity within the Iranian sample. Furthermore, the scale demonstrated positive internal validity and reliability in Persian translation. These findings suggest that the scale possesses acceptable psychometric properties, making it a valuable tool for assessing workplace fun among nurses in Persian-speaking countries.

## Introduction

Nurses play a critical role in enhancing public health by providing extensive patient care, making up 56% of hospital staff in Iran^1^. Despite their significance, their work can be highly stressful, leading to job dissatisfaction, burnout, and the desire to leave the workplace or profession^1^. However, improving the work environment for nurses may reduce job dissatisfaction, turnover, and burnout. Therefore, policymakers and health managers should take coordinated measures to address the challenges of poor work environments^2^. In recent years, there has been a growing interest in the positive effects of a lively work environment on employee behavior and performance^3^. Cultivating a fun workplace can promote constructive behavior among employees, thereby fostering creativity and innovation^4,5^. Nursing managers should consider integrating enjoyable activities into the workday to improve the work environment^3^.

Management literature emphasizes the positive impact of incorporating fun in the workplace on employees’ attitudes^3,5,6^. Organizing fun activities, such as parties, is an excellent way for organizations to show recognition to their employees^7^. Tsaur et al. study has demonstrated that engaging in enjoyable activities can help alleviate stress, foster happiness, and improve team interactions^8^. For instance, organizing social gatherings and celebrations can be an effective way for organizations to acknowledge and appreciate their employees^7^. A workplace that embraces fun can increase creativity, innovation, work performance, and employees’ emotional commitment^8^. By organizing enjoyable activities, companies showcase their dedication and support for their employees, which can enhance social engagement within the workplace^4,9^.

Workplace fun encompasses elements of the work environment that foster positive emotions and enjoyment^4^. Recreational activities are essential for organizational success and can take various forms, including social events like parties and picnics, team activities such as sports and competitions, public celebrations recognizing employee achievements, and personal milestones like birthdays and anniversaries^10^. Additionally, socializing with colleagues outside work is crucial for building friendly relationships and fostering a positive atmosphere^4,10^. Managers are vital in promoting workplace fun by emphasizing employee relationships and facilitating enjoyable activities, including games^11^. Their support enhances positive feelings, influences employees’ attitudes and behaviors, boosts intrinsic motivation and job satisfaction, and fosters creativity within the workplace^5,12^.

The impact of workplace fun on job satisfaction is significant, highlighting the necessity of a positive work environment^11^. Employees who lack opportunities for fun are at a higher risk of depression and burnout^12^. Research has consistently shown that workplace fun positively influences innovation and emotional fulfillment, particularly in nursing^5,10^. A joyful work environment contributes to nurses’ overall satisfaction and well-being, improving their attitudes and behaviors while enhancing intrinsic motivation and job satisfaction. Conversely, nurses who miss out on enjoyable experiences may experience increased stress, depression, and burnout^12^. Ultimately, those who find meaning in their work tend to be more motivated in their roles^13^.

Various researchers have sought to quantify workplace fun, with Peluchette and Karl proposing a one-dimensional framework^14^. Others have suggested one-dimensional^15^ and three-dimensional^16^ measurement scales. The four-dimensional 24-item McDowell scale is often cited as a standard criterion for assessing fun at work^17^. However, this study focuses on the multidimensional scale developed by Tews et al. due to its nuanced approach to evaluating distinct aspects of workplace fun. Tews et al. identified three key dimensions: “fun activities,” “socializing with colleagues,” and “manager support for fun.” They emphasized the importance of separately measuring these dimensions to capture their unique impacts on workplace outcomes^7^. For instance, their findings revealed that “manager support for fun” correlated negatively with turnover intention, while “fun activities” positively influenced job performance. By utilizing Tews’ scale, this study aims to provide a more comprehensive understanding of how these dimensions of workplace fun affect the nursing profession.

Despite substantial research on the advantages of integrating fun into the workplace^7,18^, there remains a need for a more comprehensive understanding of its specific effects on Iranian nurses. This pioneering study in the context of Persian-speaking countries aims to validate the psychometric properties of a tool developed by Tews et al. This tool is intended to assess the level of workplace enjoyment experienced by nurses. The primary objective of this research is to evaluate the reliability, validity, and applicability of the workplace fun scale within the nursing profession. This study’s findings can significantly improve the work environment for nurses in Persian-speaking countries by providing evidence-based recommendations for enhancing workplace fun, which could lead to increased job satisfaction and reduced turnover. Given the limited previous research on this topic in Persian-speaking countries, further exploring the scale’s relevance to Iranian nurses is crucial.

## Method

### Objective

The study’s objective was to standardize and validate the “workplace fun” questionnaire designed to assess workplace recreation and verify this scale’s structure and reliability among Iranian nurses. The process involved translating the scale into Persian, ensuring internal construct validity and reliability through multiple translations and back-translations during the initial research phase. Subsequently, the Persian adaptation of the “workplace fun” questionnaire was developed for standardization.

### Participants

The study included nurses from five educational and therapeutic hospitals in Ardabil City, northwest Iran. In line with cultural adaptation and validation studies, it is recommended that the sample size should be ten times the number of items on a scale, with a minimum accepted sample size of 200 participants. The inclusion criteria specified nurses working in a clinical role with at least six months of nursing experience without a physical or mental illness history. Nurses on continuous leave for more than three months, nursing supervisors, and managers were excluded from the study. A total of 321 clinical nurses participated in the survey, providing a larger sample size for more statistical power and increased accuracy and reliability of the results. The sample sizes from each hospital were as follows: 140 nurses from Imam Khomeini Hospital, 65 from Fatemi Hospital, 40 from Alavi Hospital, 35 from Imam Reza Hospital, and 41 from Bu Ali Hospital. Proportional stratified random sampling was used within each stratum to select participants. After obtaining ethical approval, researchers introduced the study to the nursing offices in the educational and treatment centers, explained the study’s design and objectives to the nurses, and provided guidance on completing the questionnaires. Data collection took place from January to March 2023.

### Data collection

After obtaining ethical approval, researchers presented the study to nursing offices, explained the design and objectives, and provided guidance on completing the questionnaires. Data collection took place from January to March 2023.

### Demographic characteristics form

The demographic characteristics include age, gender, marital status, education, and professional level. This data was collected from diverse participants, adding depth and breadth to the study’s findings.”

### Workplace fun scale

The Persian version of the “Workplace Fun Scale,” adapted from Tews et al., consists of 14 items across three dimensions: fun activities, coworker socializing, and manager support for fun^7^.

The original version of the workplace fun scale was assessed using fourteen items from Tews et al.‘s questionnaire^7^, administered to 297 chain restaurant workers. The original scale created by Tews et al. featured 19 items and four sub-scales: five items related to fun activities, four items about coworker socializing, five items about manager support for fun, and five questions linked to constituent attachment^7,17,19^. The factor loadings for the five fun activity items (including social events, team building activities, competitions, public celebrations of work achievements, and recognition of personal milestones) were 0.72, 0.70, 0.67, 0.63, and 0.40, respectively^7^. The constituent attachment sub-scale was removed from the central construct of workplace fun due to its mediating role, and the scale was finalized with 14 items and three sub-groups of fun activities, socializing with colleagues, and the manager’s support for fun. The rating of each item on the two sub-scales of companionship and manager support for fun was on a five-point Likert scale, from strongly disagree = 1, disagree = 2, neutral = 3, agree = 4, to strongly agree = 5. The recreation sub-scale ranged from 1 = never to 5 = always with a five-point Likert scale. The mean and standard deviation for each sub-scale were reported as follows: fun activities (2.41 ± 0.74), coworker socializing (3.76 ± 0.80), and manager support for fun (3.42 ± 0.78). The sub-scale for fun activities includes items like “I enjoy participating in fun activities at work.” In Tews et al.‘s study, the overall Cronbach’s alpha coefficient of this questionnaire was reported as 0.90, and in this study, it was reported as 0.86.

### Data analysis method

In this study, we re-evaluated the factorial structure of the three aspects of workplace fun after translating and localizing the questionnaire. To ensure the robustness of our findings, we employed structural equation modeling to test the questionnaire’s structure as a conceptual model and the partial least squares method to confirm the measurement model. The data entry and analysis were performed using SPSS version 14, LISREL version 8.8, and Smart-PLS second version.

### Face validity

Validity means that the measuring instrument can accurately measure the characteristic it is intended to measure. Face validity refers to how test questions look similar to the subject they are designed to measure. When questionnaires are translated into another language and culture, the process must be precise to maintain face validity and ensure that the translated version matches the original. In our research, we employed the method model developed by Polit and Yang. This method incorporates the principles of translation-back translation and cultural adaptation to assess formal validity. We aimed to ensure that our instruments and measures were culturally and contextually appropriate for the study population. This process allowed us to thoroughly evaluate the formal validity of our research tools, contributing to our study’s overall rigor and accuracy^20^.

### Content validity

In our research, we translated the workplace fun scale into a different language and then conducted an assessment to ensure its face validity. Following this, we determined the questionnaire’s content validity using the content validity ratio and index, as outlined in Lawshe’s model. The process entails carefully reviewing the questionnaire to ensure it effectively encompasses all the targeted ideas and structures^21^.

#### Construct validity and measurement model

A confirmatory factor analysis using the partial least squares method was performed on the Farsi version of Workplace Fun to validate the construct. The primary goal was to determine if the translated Persian version maintains the three-factor structure of the original workplace fun. Specifically, the aim was to explore the relationship between the three factors of a manager’s support for fun, companionship, and attachment, which are considered hidden variables, and their measurement through items or manifest variables in a measurement model. In the measurement model, the strength of the relationship between the factor and the observable variable (item) is shown by factor loading. Factor loadings above 0.3 are favorable, and those over 0.6 are very favorable^22^. The critical indicators utilized in the partial least squares method to assess the structural part of the model were the R2 determination coefficient and the Q2 index. The R2 value is only presented for the model’s endogenous variables, with a zero value for exogenous structures. A higher R2 value for the endogenous constructs suggests a better fit for the model^23^. Another important indicator of the model’s predictive power is the Q2 index, which determines its fit in endogenous structures. A positive Q2 value indicates a favorable fit and adequate predictive power^24^. Fit indices were employed to assess the appropriateness and validity of the model.

The chi-square index, derived by dividing chi-square by the model’s degrees of freedom, gauges the likeness of a theoretical model to the actual model. A value below 2 is desirable. A standardized residual root means square index (SRMR) below 0.05 is also desirable. Traditionally, the Goodness of Fit Index (GFI) is utilized to evaluate model fit, requiring a value of 0.9 or higher^25^. Another relevant index is the Adjusted Goodness of Fit Index (AGFI), which ranges from 0 to 1^26^ and should be equal to or greater than 0.9. The Normed Fit Index (NFI), or Bentler-Bonett index, should be above 0.9 for a sign of model fit, though some researchers believe it possesses negative bias^27^. The Tucker-Lewis index (TLI) and Comparative Fit Index (CFI) are deemed acceptable above 0.9, indicating the model’s appropriateness^27^.

### Discriminant validity & divergent

The concept of discriminant validity is essential for ensuring that measures of different factors are distinct. In surveys or questionnaires, it’s crucial to ensure that the questions related to each factor are unique and don’t overlap. It is essential for evaluating measurement models in PLS (partial least squares) based on factor loadings linked to each construct’s items^28^. This study rigorously assessed divergent validity using two robust methods: the Fornell-Larcker matrix and the heterotrait monotrait (HTMT) index. According to Fornell and Larcker (1981)^29^, divergent validity is achieved when the average variance extracted (AVE) for each construct exceeds the shared variance between that construct and other constructs (i.e., the square of the correlation coefficients between constructs) in the model. According to this, the data indicates that a specific element within the model has a stronger correlation with its indicators than its relationship with other elements. In partial least squares and structural equation modeling context, divergent validity is assessed using a matrix containing the correlation coefficients between the constructs, with the square of the AVE placed along the main diagonal. The HTMT index, introduced by Hensler et al. (2015)^30^, has replaced the Fornell-Larcker method for validity assessment. According to this criterion, divergent validity is satisfactory when the HTMT index is less than 0.9. This study manually calculated the HTMT index using a formula^31^.

### Convergent validity

Convergent validity is a measure that indicates how closely related the measurement items within a category are. It’s essential to ensure that a questionnaire accurately measures what it intends to measure. This study used two leading indicators to assess convergent validity: average variance extracted (AVE) and the composite reliability index (CR). The AVE indicates the average variance shared between each construct and its indicators, with a higher AVE suggesting a better fit^32^. According to Fornell and Larcker (1981), convergent validity is achieved when the AVE exceeds 0.5^29^. The second indicator, CR, demonstrates convergent validity when greater than 0.7 and higher than the AVE (CR > AVE). Meeting these conditions ensures the presence of convergent validity^33^.

### Reliability indicators

- Internal consistency reliability was evaluated using Cronbach’s alpha and Composite Reliability (CR). The CR was reported in both the reliability and concurrent validity sections as it indicates the reliability of constructs based on their inter-correlation.
- Test-Retest Reliability: A subset of 40 participants from the original sample was re-evaluated to assess stability over time.

## Results

### Demographic characteristics of the studied sample

Three hundred twenty-one nurses from five hospitals in Ardabil participated in the study. The average age of the participants was 34.28 years (SD = 7.05), with an average total work experience of 10.48 years (SD = 7.03) and an average work experience in their current unit of 6.20 years (SD = 5.64). The majority of the participants were married (219, 68.2%), held a bachelor’s degree (285, 88.8%), and were employed as nurses (291, 90.7%). Additionally, 92 participants (28.6%) worked in medical units (Table 1).

**Table 1:** Socio-demographic characteristics of the participants (*N* = 321).

Variables	Categories	Mean ± SD	
Age (year)		34.28 ± 7.05	
Work experience		10.48 ± 7.03	
Work experience in the current unit		6.20 ± 5.64	
		Percentage	No.
Gender	Male	21.8	70
	Female	78.2	251
Marital status	Single	31.8	102
	Married	68.2	219
Employment status	Employed nurses	90.7	291
	Contract nurses	9.3	30
Educational level	Associate degree & Master’s degree and PhD	11.2	36
	Bachelor’s degree	88.8	285
Work in medical units		28.6	92

### Descriptive statistics of the three subscales of the studied questionnaire

The research scales were meticulously analyzed using descriptive statistics, such as the mean and standard deviation. The mean and standard deviation for factors 1 to 3 (Fun activities, Coworkers socializing, and manager support for fun) were precisely calculated to be 2.125 (± 0.95), 3.455 (± 0.82), and 3.272 (± 0.81), respectively. This is the lowest mean related to the Fun subscale activities and the highest mean corresponding to the subscale Coworkers were socializing.

**Table 2:** Descriptive indices of research variables & indices of the convergent and discriminant validity and re-ability of the workplace-fun inventory (*n* = 321).

Subscale	Mean	SD	*R* ^2^ Weak 0.19 Medium 0.33 Strong 0.67	Q^2^ Weak 0.02 Medium 0.15 Strong 0.35	AVE < 0.5	Re-test coefficients	(α)Cronbach’s Alpha > 0.7	Composite reliability (CR) > 0.7
Admin fun	2.125	0.951	0.258	0.193	0.655	0.361*	0.822	0.885
Colleague socializing	3.455	0.829	0.604	0.427	0.766	0.770**	0.923	0.943
Constituent attachment	3.272	0.817	0.603	0.326	0.595	0.705**	0.828	0.992
Workplace fun	2.951	0.627				0.459**	0.859	0.885

*p* < 0.05*,*p* < 0.01**.

### Face validity

The original version of the fun at work questionnaire, a pivotal starting point for our research, was created by Tews et al.^7^. With permission from the original designers and following the principles of translation-back translation, two bilingual experts in English and Farsi undertook the translation and comparison. This process was then repeated by two additional translators, who were unaware of each other’s work, to ensure the accuracy of the translation. The resulting translations were combined, and the most appropriate wording was selected. In the final step, two more translators, also unaware of the original text, verified the correspondence of the translated text with the original questionnaire^19^.

### Content validity

The content validity of our research was rigorously validated using Lawshe’s model. We involved 14 experts and professors from the Ardabil University of Medical Sciences nursing education field to assess the necessity of items using a three-point Likert scale. The high validity ratio of 0.99, based on the number of evaluators, indicates appropriate validity. Additionally, we calculated the CVR index for each of the 14 items, representing the ratio of evaluators who considered an item necessary or valuable to the total number of evaluators. Furthermore, we utilized the CVI/Universal S—index to gauge the relevance of each item. A high percentage of items received scores of 3 and 4 from all experts, indicating acceptable content validity (0.8 or higher)^34^. All the items scored above 3 in our study, confirming their appropriate content validity.

### Construct validity

To validate the construct, we utilized the second-order confirmatory factor analysis method. This approach allowed us to investigate the relationship between the observable and latent variables and the relationship between the latent variables and the original construct. Initially, we focused on the three subscales of fun at work:

Manager’s support for fun (items 1 to 4).

Socializing with colleagues (items 5 to 9).

Attachment to the structure (items 10 to 14).

**Fig. 1:**
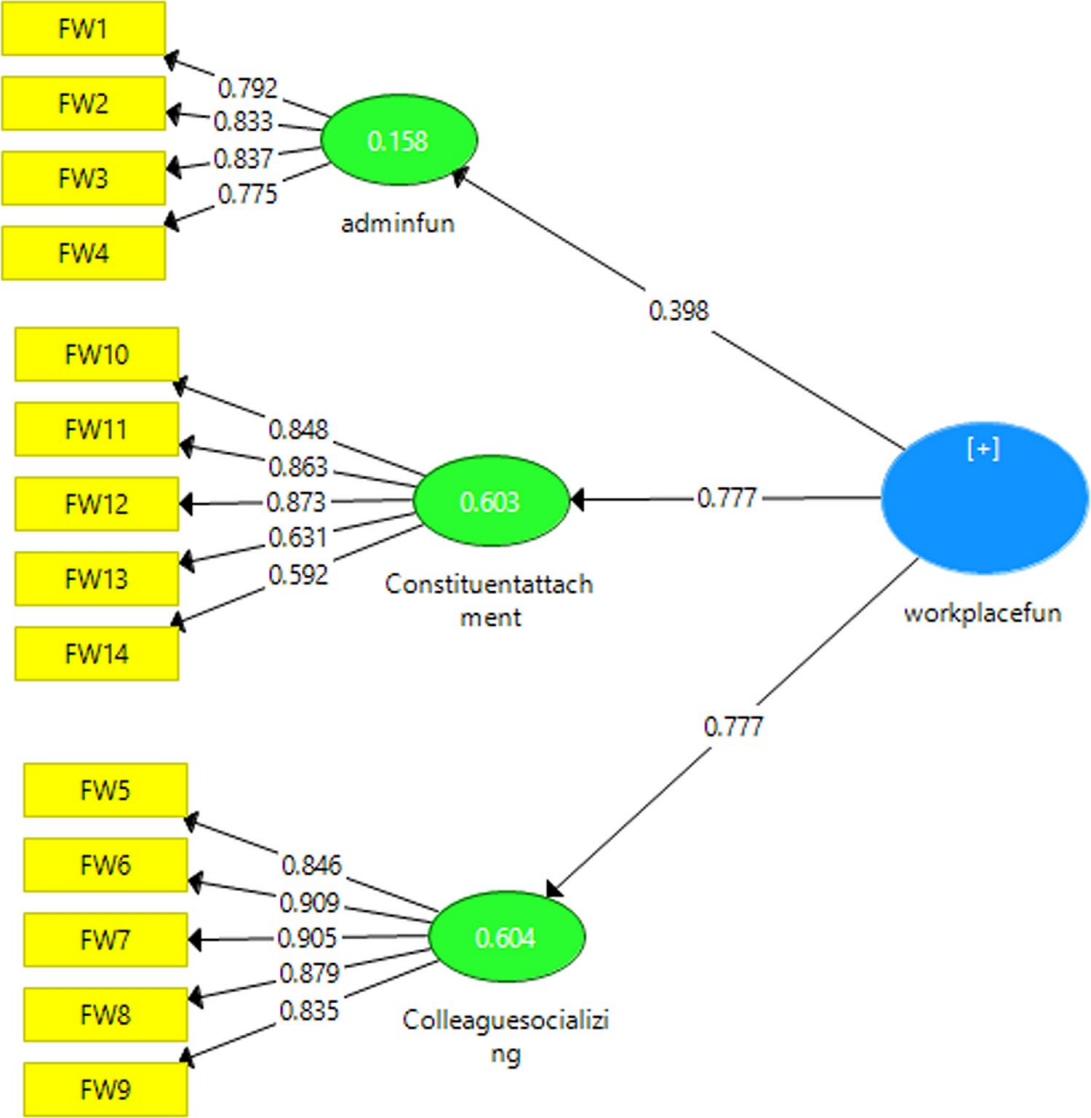
Model for measuring the concept of fun at work.

We assessed the relationship between the latent variables and items through a measurement model. The results in Table 2, with factor loadings of all 12 questions above 0.6, demonstrate a robust relationship between items and factors, thus confirming the construct. In Fig. 1, ellipses represent latent variables or factors, and rectangles represent the workplace fun questionnaire items. Arrows from ellipses to rectangles show which factor the items load on, and the values written on the arrows show the amount of variance of the items that the factor can explain. The R2 values falling within the acceptable range, as reported in Table 2, indicate a satisfactory fit of the model, and the Q2 values, also within their acceptable range, indicate the model’s good predictability in three endogenous variables (Table 2).

The measurement model for workplace fun, as presented in Table 3, demonstrates strong validity across multiple indicators. The ratio of chi-square to the degree of freedom is 1.27, which is favorable as it is less than 2. Furthermore, the GFI, AGFI, CFI, NFI, and TLI indices are 0.97, 0.94, 0.99, 0.98, and 0.97, respectively, surpassing the threshold of 0.9. The SRMR index is 0.04, below 0.05, further affirming the excellent fit of the measurement model. The workplace fun measurement model demonstrates a suitable and acceptable fit in the Iranian sample, supporting (Table 3).

**Table 3:** Goodness of fit indicators of the measurement model of the workplace-fun.

Model indicators	X^2^/df	GFI	AGFI	CFI	NFI	TLI	SRMR
Measurement pattern	1.27	0.97	0. 94	0.99	0.98	0.97	0.04

### Discriminant and divergent validity

Our study used the Fornell-Larcker matrix and HTMT index to assess divergent validity. The Fornell-Larcker matrix showed that the numbers on the main diagonal were higher than those below, confirming the model’s validity. The HTMT criterion, with an acceptable limit of less than 0.9, was met, indicating acceptable divergent validity. The values of this index for all three subscales were below 0.9, demonstrating that the subscales are distinct (see Table 4).

**Table 4:** Fornell and Larcker matrix, Divergent & discriminant validity indices of workplace-fun scale.

Subscales	Admin fun	Colleague socializing	Constituent attachment
Admin fun	**0.810**		
Colleague socializing	0.240 *0.258*	**0.875**	
Constituent attachment	0.390 *0.453*	0.272 *0.301*	**0.771**

In each cell, the upper numbers indicate the correlation coefficient and the lower numbers indicate the values ​​of the HTMT index. The numbers on the matrix diameter are bolded.

### Convergent validity

The research examined convergent validity using average variance extracted (AVE) and composite reliability. According to Fornell and Larcker, convergent validity is present when AVE exceeds 0.5. In this study, the AVE values for all three constructs are higher than 0.5, confirming the convergent validity between the constructs and their items. Additionally, convergent validity is confirmed when the composite reliability (CR) is higher than 0.7 and when CR is greater than the average variance extracted (CR > AVE). Meeting these criteria establishes convergent validity. The values in the table for CR are all above 0.7 and more significant than the AVE values in each relevant subscale, confirming the establishment of convergent validity (Table 2).

### Scale reliability

#### Internal consistency reliability

The internal consistency of the ‘workplace fun’ instrument was assessed using Cronbach’s alpha coefficient. The overall Cronbach’s alpha for the entire instrument was 0.859, indicating acceptable internal consistency, as values above 0.7 are considered satisfactory. The alpha coefficients for the first, second, and third factors were 0.822, 0.923, and 0.828, respectively, demonstrating suitable internal consistency for the test questions. The CR composite coefficient was used to assess the reliability of the ‘workplace fun’ scale. The combined reliability coefficients for factors 1 to 3 and the overall factor were 0.885, 0.943, 0.992, and for workplace fun 0.885, respectively – all greater than 0.7, indicating high reliability for both the subscales and the overall factor. This high reliability should make you feel secure in the use of the ‘workplace fun’ scale (Table 2).

#### Test-retest reliability

To further underscore the stability of the test over time, a retest was conducted with 40 participants from the original sample after two weeks. The retest correlation coefficients for factors one to three and the total score of the questionnaire were 0.361, 0.770, 0.707, and 0.459, respectively. These coefficients were significant at the level of 0.022, 0.001, 0.001, and 0.003, respectively; they provide strong evidence of the robust stability of the instrument over time and indicate the stability of the results in the long term.

## Discussion

The study aims to assess the psychometric properties of the Persian version of the Workplace Fun Scale among nurses. The Workplace Fun Scale, initially developed by Tews et al.^7^, is being adapted to the Iranian cultural context. Given the high-stress nature of nursing, it is crucial to understand how fun and enjoyment in the workplace can contribute to a positive work environment. The primary objective of this study is to evaluate the reliability and validity of the Persian version of the Workplace Fun Scale, ensuring its effectiveness in measuring workplace fun among Persian-speaking nurses.

### Validity results

The Persian version of the Workplace Fun Scale has received strong support from the confirmatory factor analysis (CFA) results. All items showed significant factor loadings exceeding 0.6. The chi-square to degrees of freedom ratio was calculated at 1.27, indicating a favorable fit for the model. Additionally, the goodness-of-fit indices were impressive, with GFI at 0.97, AGFI at 0.94, CFI at 0.99, NFI at 0.98, and TLI at 0.97, all exceeding the acceptable threshold of 0.9. These findings confirm that the scale effectively captures the construct of workplace fun among nurses.

Furthermore, based on expert evaluations, the content validity ratio (CVR) of 0.99 confirms that almost all items are necessary for measuring workplace fun, enhancing the tool’s relevance in the Iranian context. The confirmatory factor analysis results on Iranian nurses in the present study supported the three-factor model. The optimal values of the indicators in the factor analysis showed that the hidden variables or the three translated subscales effectively measured the manifest variables or questions. This finding was consistent with the results obtained in the study by Jing et al.^5^. In their research, et al. utilized the Chinese translation of the 14-question, three-factor Tews scale, which identified three key factors: fun at work, socializing with colleagues, and manager support for fun. Our data similarly demonstrated a strong correlation with these factors, particularly in fun at work and manager support, reinforcing the validity of Jing et al.‘s findings in different contexts. This alignment suggests that the elements contributing to workplace enjoyment are consistent across diverse populations.

Additionally, the convergent and discriminant validity assessment revealed that the average variance extracted (AVE) for each factor exceeded the 0.5 threshold, confirming the meaningful contribution of the items to their respective constructs. According to the Fornell-Larcker criterion, the correlations between subscales were lower than the square root of their AVE values, further validating the distinctiveness of each dimension. This comprehensive approach to validity ensures that the Persian version of the Workplace Fun Scale is culturally appropriate and methodologically sound. Furthermore, these findings align with previous studies, such as those by Jing et al.^5^ and Müceldili & Erdil^18^, reinforcing the scale’s applicability across various cultural contexts, including the Iranian nursing environment.

### Reliability results

The reliability analysis yielded strong results, with an overall Cronbach’s alpha coefficient of 0.859, indicating excellent internal consistency for the scale. Each subscale also demonstrated high reliability, with coefficients of 0.822 for fun activities, 0.923 for coworkers socializing, and 0.828 for manager support for fun, all surpassing the acceptable threshold of 0.7. The test-retest reliability over two weeks revealed significant correlation coefficients, confirming the scale’s stability over time. These findings are consistent with prior research by Tews et al.^7^ and Müceldili et al.^18^, which reported strong reliability coefficients for their versions of the same scale. Furthermore, Cronbach’s alpha for the three subscales in Tews et al.‘s 14-item scale were 0.85, 0.86, and 0.93^18^. Another study by Tews et al.^35^ on the impact of fun in the workplace and training climate showed high internal consistency for the three subscales of types of fun, support from the manager, and hanging out with friends, with reliability coefficients of 0.66, 0.76, and 0.77, respectively^35^. Similarly, Jing obtained Cronbach’s alphas of 0.89, 0.81, and 0.82 for these three subscales^5^. All three studies indicated favorable reliability for each subscale.

In this study, the researchers utilized convergent validity and traditional reliability measures to ensure the accuracy of their findings. The high composite reliability coefficients indicate that the scale’s measurements were consistent and confirm that the Persian version of the Workplace Fun Scale is reliable for assessing workplace fun among nurses. These findings are particularly significant for healthcare administrators, as they can use this scale to pinpoint areas for improvement in workplace dynamics, potentially leading to increased job satisfaction and employee retention. The study also emphasized various dimensions of workplace fun, highlighting the need for prompt and targeted interventions to address different aspects of fun at work, in line with existing literature^35,36^. Additionally, the scale consistently measured each dimension and demonstrated favorable reliability over time. These results align with previous studies that found high reliability in similar scales.

This study represents the first attempt to measure and validate the Workplace Fun Scale specifically for nurses in Iran. It builds on quantitative analyses of workplace recreation in the United States^7,17,35,37^. Workplace fun encompasses entertainment programs, socializing with colleagues, and managerial support. The practical implications of this research are significant, as it provides a roadmap for hospital managers to enhance employees’ well-being and satisfaction by introducing enjoyable activities and fostering a supportive environment. This approach can motivate employees to engage positively with each other and encourage positive changes instead of maintaining the current situation^36^. Future studies should consider cultural differences in workplace enjoyment to develop tools relevant to specific contexts.

### Limitations

It is important to note that there are certain limitations to the study. Firstly, the workplace fun scale was translated and evaluated based on its three-factor structure through confirmatory factor analysis. However, this approach needs to be revised using qualitative methods while considering the cultural needs of the recreation factor. Secondly, a native questionnaire was developed, which should be analyzed using exploratory factor analysis. Lastly, the research was limited to nurses and healthcare workers in Ardabil, Iran. Therefore, more research is urgently needed to generalize the validation outcomes and explore the concept of fun at work across different cultures and professions. The following area is crucial and requires additional investigation.”

## Conclusion

The confirmatory factor analysis results for the ‘Workplace Fun’ scale, developed by Tews et al. for an Iranian sample, revealed highly positive outcomes. The study indicated that the questions’ structure and internal consistency were consistent with the original scale and well-suited for the Iranian sample. The results suggest that the Persian-translated scale exhibits strong internal validity and reliability. These findings indicate that the scale possesses acceptable psychometric properties, making it a dependable tool for measuring the intended constructs. These findings imply that the scale can be effectively and accurately employed in Persian-speaking populations. Consequently, it can confidently measure the ‘workplace fun’ level in the Iranian context.

## Data Availability

The data that support the findings of this study are available from the corresponding author [A. M.] upon request.
